# Nano-Architecture of Persistent Focal DNA Damage Regions in the Minipig Epidermis Weeks after Acute γ-Irradiation

**DOI:** 10.3390/biom13101518

**Published:** 2023-10-13

**Authors:** Harry Scherthan, Beatrice Geiger, David Ridinger, Jessica Müller, Diane Riccobono, Felix Bestvater, Matthias Port, Michael Hausmann

**Affiliations:** 1 Bundeswehr Institute for Radiobiology Affiliated to the University of Ulm, Neuherbergstr. 11, D-80937 München, Germanymatthiasport@bundeswehr.org (M.P.); 2 Kirchhoff-Institute for Physics, Heidelberg University, Im Neuenheimer Feld 227, D-69120 Heidelberg, Germanydr.research@mailbox.org (D.R.); 3 Département des Effets Biologiques des Rayonnements, French Armed Forces Biomedical Research Institute, UMR 1296, BP 73, 91223 Brétigny-sur-Orge, France; diane.riccobono@def.gouv.fr; 4 Core Facility Light Microscopy, German Cancer Research Center (DKFZ), Im Neuenheimer Feld 280, D-69120 Heidelberg, Germany; f.bestvater@dkfz-heidelberg.de

**Keywords:** 53BP1, ATM, cutaneous radiation syndrome, DNA damage response, γ-H2AX, γ-irradiation, MRE11, persistent DNA damage, pig skin, persistent homology, Ripley statistics, Single Molecule Localization Microscopy (SMLM)

## Abstract

Exposure to high acute doses of ionizing radiation (IR) can induce cutaneous radiation syndrome. Weeks after such radiation insults, keratinocyte nuclei of the epidermis exhibit persisting genomic lesions that present as focal accumulations of DNA double-strand break (DSB) damage marker proteins. Knowledge about the nanostructure of these genomic lesions is scarce. Here, we compared the chromatin nano-architecture with respect to DNA damage response (DDR) factors in persistent genomic DNA damage regions and healthy chromatin in epidermis sections of two minipigs 28 days after lumbar irradiation with ~50 Gy γ-rays, using single-molecule localization microscopy (SMLM) combined with geometric and topological mathematical analyses. SMLM analysis of fluorochrome-stained paraffin sections revealed, within keratinocyte nuclei with perisitent DNA damage, the nano-arrangements of pATM, 53BP1 and Mre11 DDR proteins in γ-H2AX-positive focal chromatin areas (termed macro-foci). It was found that persistent macro-foci contained on average ~70% of 53BP1, ~23% of MRE11 and ~25% of pATM single molecule signals of a nucleus. MRE11 and pATM fluorescent tags were organized in focal nanoclusters peaking at about 40 nm diameter, while 53BP1 tags formed nanoclusters that made up super-foci of about 300 nm in size. Relative to undamaged nuclear chromatin, the enrichment of DDR protein signal tags in γ-H2AX macro-foci was on average 8.7-fold (±3) for 53BP1, 3.4-fold (±1.3) for MRE11 and 3.6-fold (±1.8) for pATM. The persistent macro-foci of minipig epidermis displayed a ~2-fold enrichment of DDR proteins, relative to DSB foci of lymphoblastoid control cells 30 min after 0.5 Gy X-ray exposure. A lasting accumulation of damage signaling and sensing molecules such as pATM and 53BP1, as well as the DSB end-processing protein MRE11 in the persistent macro-foci suggests the presence of diverse DNA damages which pose an insurmountable problem for DSB repair.

## 1. Introduction

Localized high-dose and high-dose-rate ionizing radiation (IR) exposures as noted in accidents and in radiotherapy, can lead to a dose-dependent cutaneous reaction that involves inflammation and erythema as well as long-term effects such as fibrosis, keratosis and skin cancer [1,2]. Accidental acute high-dose IR exposures often induce localized radiation burns and ulcerations [3,4,5]. While there is ample knowledge about the cutaneous radiation response after radiotherapy or accidents [6,7,8,9,10,11], the DNA damage response after acute high-dose exposure in the skin of large animal models has so far been studied in pigs [12,13,14].

Ionizing radiation induces DNA damage of which the DNA double-strand break (DSB) lesion is the most severe threat to cellular survival and genome integrity. In surviving cells, erroneous DNA repair may lead to stochastic effects that result from error-prone DNA repair pathways thereby fueling cancer development. DSBs induce a complex DNA damage response (DDR) that usually persists up to the completion of repair or cell death [15,16,17]. DSBs are preferentially repaired by the non-homologous end joining (NHEJ) pathway(s) and homology-directed repair (HDR) [18,19]. Moreover, error-prone PARP1-dependent alternative NHEJ may contribute to the processing of complex DNA damage that is abundant after high linear energy transfer (LET) irradiation [18]. DSBs in the late S and G2/M phases are repaired by NHEJ and HDR, the latter being considered error-free when a template for homologous recombination repair is available [15,20].

DSB ends are bound by sensor proteins like Ku (NHEJ) or MRN (HDR), with the latter activating the apical ATM kinase that, among other targets [21], phosphorylates histone H2AX on serine 139 (then called γ-H2AX) in the chromatin domain surrounding a DSB [22] leading to microscopically visible nuclear foci. Each γ-H2AX focus represents at least one DSB in low-dose irradiation scenarios [23,24], while at higher doses or after high LET particulate irradiation more than one DSB and multiple DNA damage types can be found within a DNA repair focus [25,26,27,28,29]. After DSB repair, γH2AX molecules are dephosphorylated or turned over, leading to the disappearance of radiation-induced foci [30,31,32,33]. However, a subclass of radiation-induced foci may persist up to days or even weeks and are likely the result of complex DNA damage that hinders completion of repair and stimulates repair attempts [13,28,34,35,36,37]. In some cases, γ-H2AX foci may remain in chromatin even after DSB endjoining [36,38,39]. Besides, γH2AX DSB foci have been noted up to 7 days post-exposure in irradiated mouse skin and may serve as a biodosimeter in accident scenarios [40]. Similar observations have been reported for the 53BP1 DNA damage sensor protein [8,28,41] that also accumulates in the chromatin domain around DSBs [42,43,44] and instigates and directs the DNA damage response [45,46]. Persistent DSB foci have been noted in a variety of acute and high LET exposure scenarios but their significance remains a matter of debate [47]. It has been assumed that persistent foci are a consequence of complex DNA damage that can only be repaired with delay, or not at all, and thus persist or induce cell death [27,36,48]. They may even be passed on to daughter cells [38,49] and/or seem to be involved in checkpoint signaling of damaged or altered chromatin structures [28,50,51]. In addition, lack of dephosphorylation may contribute to the persistence of DSB-indicating foci in chromatin subcompartments [31,32,33], and irreparable DNA damage has been implied in the senescence-associated secretory phenotype (SASP) in radiation induced senescence [52,53]. 

During the last decade the spatial organization of DDR proteins within DSB repair foci on the nanoscale has been increasingly investigated by super-resolution single-molecule localization microscopy (SMLM) (for review see [54]), a microscopic technique by which the coordinates of single fluorescence labeling molecules can be determined within a cell nucleus with a precision in the ten-nanometer range [55]. Coordinate matrices of single-molecule signals are the basis for further mathematical calculations of geometry and topology, e.g., Ripley distance frequency statistics of pairwise single molecule signal distances, cluster formation algorithms and persistence homology analyses [56].

Such approaches have revealed that ionizing photon or particle irradiation-induced DSBs lead to rearrangements of the chromatin nano-organization in relation to repair processes at given damage sites. Broken DNA strands in heterochromatin lead to heterochromatin relaxation [57] with the DSB ends being transferred to the border of densely packaged heterochromatin regions [58,59] likely by entropic forces [60]. SMLM revealed that γ-H2AX clusters encompassing DSBs are equally sized on average [61], and furthermore, display a high topological similarity, especially when the γ-H2AX clusters are situated in heterochromatin regions [62]. Usually, this similarity of γ-H2AX cluster nano-topology is generally higher early (~30 min) after irradiation than at later time points (e.g., 24 h), which indicates that γ-H2AX clusters and the underlying chromatin domain are relaxed after completion of DSB repair. Some clusters, however, persist over longer time periods while maintaining an early topological organization [63], which may indicate the presence of damage that obstructs repair progression.

So far, knowledge of the nano-organization of persistent chromatin regions with DSB damage after high-dose irradiation is scarce, especially in tissue context. A previous study on stem cell treatment options for the cutaneous radiation syndrome exposed lumbar skin regions of Göttingen minipigs to ~ 50 Gy of Co-60 γ rays [64]. Several weeks after irradiation it was noted that the previously irradiated epidermal keratinocytes still harbor persistent DSB-related foci [13]. Here, we utilized paraffin-embedded tissue of minipig skin samples 28 days after 50 Gy ^60^Co γ-irradiation to study the nano-organization of the persistent DSB-related focal damage in keratinocyte nuclei by SMLM using the DDR protein markers 53BP1, MRE11 and activated phospho-S1981-(p)ATM. We particularly addressed the nano-organization of these markers within γ-H2AX-marked regions (macro-foci) and relative to undamaged chromatin. Moreover, we compared the minipig data with freshly induced simple DSB regions of GM12878 lymphoblastoid control cells. The data obtained suggest that persistent DNA damage foci of keratinocytes likely represent chromatin regions that are refractory to DSB repair.

## 2. Materials and Methods

### 2.1. Experimental Minipig Radiation Model and Cell Lines

The tissues analyzed in this study were from the experimental Göttingen minipig model studies described previously [64]. For this study, paraffin-embedded formaldehyde-fixed skin biopsies from the radiation source-proximal lumbar skin region “A” of Agay et al. (cf. Figure 2 in Ref. [64]) from two female Gottingen Minipigs were used. The lumbar skin had been exposed to 50.6 ± 4.1 Gy gamma irradiation with a 60 Co γ source (IRDI 4000; Alstom) at a dose rate of 0.6 Gy min^−1^. The doses will reflect the surface dose absorbed by the epidermal regions studied [64]. Skin samples of two pigs (P201, P212) 28 days post-irradiation were chosen for this study, as their keratinocyte nuclei display persistent DNA damage foci [13]. 

All animal trials were approved by the Animal Ethics Committee of the French Armed Forces Biomedical Research Institute (N°2008/24.0). All pigs were handled in compliance with the French legislation related to animal care and protection [64]. 

For comparison, we used the lymphoblastoid cell line (LCL) GM12878 (Coriell Institute, Camden NJ, USA) as a control, as it serves as a positive control in all our immunofluorescent staining experiments. An LCL cell suspension was exposed to 0.5 Gy X-rays using a Maxishot SPE X-ray cabinet (Yxlon, Hamburg, Germany) at 240 kV, 13 mA, 1 Gy/min (water kerma) followed by incubation for 30 °C in an incubator at 37 °C and fixation in 70% ethanol [65]. This dose was chosen as it induces well-separated single DSB foci. The specificity of the antibodies used for pig and human cells was demonstrated in previous investigations [65,66].

### 2.2. Immunofluorescence and Image Analysis

Paraffin skin tissue sections (8 μm) of the skin biopsies of day 28 post-irradiation were mounted on super-frosted slides and processed and immunostained as described in detail elsewhere [13,29], with the exception that immunostaining was conducted in TCTG buffer (TRIS, 1% Na-Casein, 0.1% Tween20, 0.1% fish-gelatin, pH 7.3–7.4) to reduce background. The slides were incubated with the primary antibodies for 1 h at 37 °C in TCTG buffer followed by three 5 min washes in TCTG and incubation with the secondary antibodies for 1 h at 37 °C. The antibodies, their sources and the dilutions used are listed in Table 1. After incubation with the secondary antibodies, sections were washed (3 × 5 min) in TCTG at 37 °C. Slides were supplied with 18 μL Prolong Gold Mounting Medium (Life technologies, Thermo Fisher Scientific, Darmstadt, Germany) containing DAPI (4′,6-diamidino-2-phenylindole) as DNA/nuclear counterstain and covered with a 24 × 60 mm cover slip. Preparations were cured for 2 days at RT and subjected to SMLM as described previously [29]. Widefield images were recorded using the ISIS fluorescence imaging system (MetaSystems, Altlussheim, Germany) equipped with an Axioimager 2i and 63× lens (both Zeiss, Oberkochen, Germany).

### 2.3. Statistical Analysis

The local positions of the signal tags were obtained from the registration of molecular blinking events. The coordinates of these points were acquired in a so-called “Orte-Matrix”, which also contained information about the signal amplitude, position errors, etc. On the basis of this “Orte-Matrix” all further evaluation procedures were processed.

The distribution of signal tags throughout nuclei was analyzed using the in-house developed program for cluster analysis based on DBScan [67] and Ripley’s K statistics based on a frequency distribution of pairwise distances of the investigated proteins [56]. From these curves organizational structures in the protein distribution can be obtained. The cluster analysis built upon the DBSCAN algorithm detects clusters of signal tags and provides information about their characteristics, such as the number of clusters per nucleus and the percentage and density of signals within clusters and outside of clusters, i.e., the non-clustered signal in the surrounding chromatin.

### 2.4. Analysis of Persistent Homologies

Persistent homologies [62,68,69] calculated for the signal tag patterns in the different γ-H2AX outlined foci allow a scale and rotational invariant definition of characteristic shape-related topological parameters. Here, we applied dimension 0 (components) and dimension 1 (holes) as values for comparison of cluster formations. In algebraic topology, these so-called Betti numbers for zero- and one-dimensional simplicial complexes are the topological invariants that can be compared. As a measure for similarities of clusters, the Jaccard index was calculated [70] and visualized in heatmaps. This normalized similarity measure is a value between 0 and 1, where a value of 0 means no similarity and 1 is the identity [56]. 

## 3. Results

**Figure 1:**
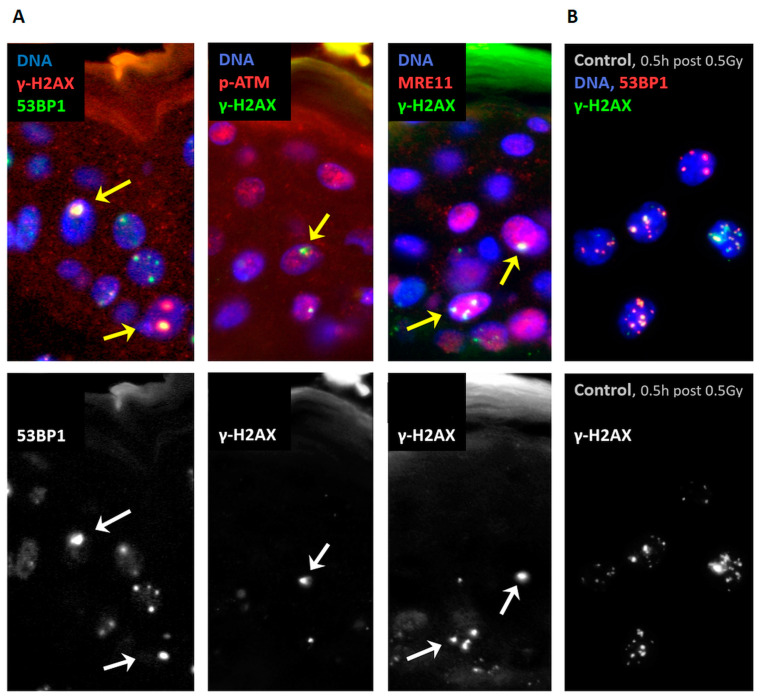
(**A**) Wide-field fluorescence images of paraffin sections of minipig skin stained for persistent nuclear (DAPI, blue) foci (arrowed) displaying the DSB damage markers 53BP1, MRE11, pATM (detected with Cy3, red) and co-stained for γ-H2AX (Alexa488, green; for color coding see upper left corner of the image details). The keratin layer of the skin (orange/green) is situated at the top of the image details. Persistent foci are absent from non-irradiated skin (see Figure 2 in ref. [13]). (**B**) Image of control LCL cells 30 min after 0.5 Gy X irradiation displaying colocalizing foci for γ-H2AX and 53BP1. Grayscale images in the lower row show the green γ-H2AX channel of the RGB figure for better color discrimination.

Acute irradiation of tissues leads to DSB damage, most of which is repaired rapidly. However, genomic regions that contain complex DNA damage may be inhibitory to repair and remain present as persisting foci, which has been noted in minipig epidermal keratinocytes up to several weeks after the radiation insult. Here, we investigated paraffin tissue sections of lumbal skin of two minipigs 28 days after 50 Gy γ-irradiation that contain cells with persistent DSB-related focal damage regions [13] that were revealed by widefield microscopy after immunostaining with γ-H2AX, 53BP1, MRE11 and activated phospho-ATM (pATM) antibodies (Figure 1A). We additionally compared the minipig results to those of freshly induced DSB lesions in nuclei of lymphoblastoid cell line (LCL) control cells 30 min after 0.5 Gy X-ray exposure (Figure 1B).

### 3.1. Single Molecule Localization Analysis of DDR Proteins in Nuclei with Damage

**Figure 2:**
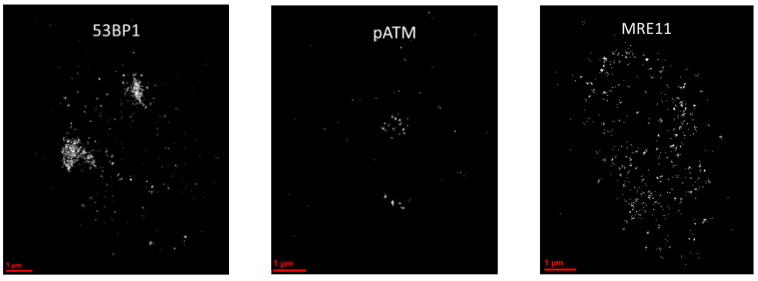
SMLM images of the Cy3 channel as illustrations of the structures. White dots represent clusters of signal tag blinking events. 53BP1 signal tags form super-clusters across the areas (foci) of DNA damage. pATM is restricted to small parts of the DNA damage areas, while MRE11 forms numerous clusters inside the γH2AX-tagged regions and was also dispersed across the entire nuclear chromatin.

To further investigate the chromatin organization within the persistent DDR foci in minipig cells, we developed a protocol for SMLM analysis in paraffin-embedded skin sections. It was observed that the clear immunofluorescent signals obtained in paraffin sections could be successfully analyzed by SMLM, yielding data sets of pointillist marker distributions that were well suited to mathematical operations. In SMLM of tissue sections, all DDR markers performed well, except for γ-H2AX which, albeit showing clear signals of DSB foci in the widefield microscope, failed to engage in the blinking events required for SMLM. Thus, we choose γ-H2AX as a widefield marker to select regions of interest (ROIs) tagging persistent DNA damage regions within keratinocyte nuclei of the irradiated epidermis. Within such γ-H2AX-defined ROIs (termed macro-foci) we measured the blinking events of the 53BP1, MRE11 and pATM single molecule tags (Figure 2). Visual inspection of typical pointillist images revealed the different characteristics of the three DDR proteins in terms of their spatial nano-distribution. While MRE11 was dispersed over the whole nucleus with only forming small nanoclusters within macro-foci, 53BP1 accumulated in large clusters (often overlapping with γ-H2AX macro-foci) with only a few protein signals dispersed throughout the undamaged nuclear chromatin. Activated phospho-Ser-1981-(p)ATM single molecule signals were confined to macro-foci as small nanoclusters (Figure 2).

**Figure 3:**
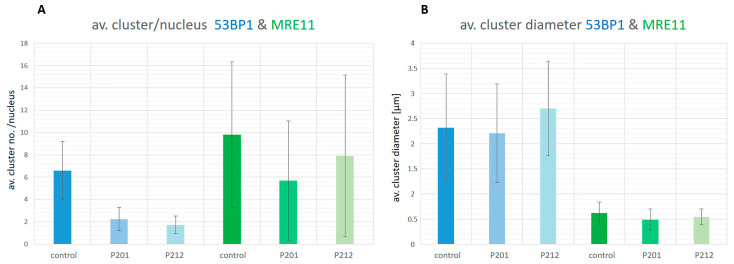
(**A**) Average number of super-clusters of nano-signal tags per nucleus (*n* = 25) in LCL controls and minipig P201 and P212 skin samples. For 53BP1 (blue bars) there were on average 6.3 super-clusters containing nano-signal tags per 0.5 Gy X-irradiated LCL (control; 30min post-IR) nucleus, while the minipig keratinocytes with clusters displayed ~2 super-clusters per nucleus, a significant difference (*p* < 0.0001). For MRE11 (green bars) there were ~10 SMST clusters per nucleus in control cells and about 6 or 8 clusters per nucleus in the two minipigs, a significant difference between LCL control and minipig P201 cells (*p* = 0.0199). The rare and small nanoclusters of the pATM signal tags failed to reveal super-clusters. (**B**) Average diameter of super-clusters of nanotags per cell nucleus in LCL controls and minipig P201 and P212 skin samples. The average 53BP1 and MRE11 super-cluster diameters were similar similar in minipig and LCL control cells.

Based on these qualitative results we performed statistical comparisons regarding cluster formation and the signal distribution and density relative to undamaged nuclear chromatin and between samples. First, we determined the average numbers of 53BP1 and MRE11 single molecule signal tag (SMST) clusters per nucleus in the minipig skin samples and the control LCL cells (Figure 3A). 53BP1 SMST clusters (defined by >54 nano signal tags within a 200 nm search radius) were observed in all minipig samples. LCL control nuclei 30 min post 0.5 Gy X-irradiation on average displayed 6.6 (±2.6) 53BP1 single molecule signal super-clusters (corresponding to macro-foci the widefield microscope) per nucleus. The persistent focal damage-carrying keratinocytes of the minipig epidermides, on the other hand, displayed on average 2.2 (±1.1) SMST super-clusters per keratinocyte nucleus in minipig P201 and 1.7 (±0.8) in P212 twenty-six days post-irradiation (Figure 3), with the difference between minipig and control samples being significant (*p* < 0.0001). The super-cluster numbers in the minipigs match the average focus number observed previously by widefield microscopy [13].

Measuring the diameter of the SMST clusters (Figure 3B) showed that 53BP1 formed large super-clusters of about 2.5 µm, which correspond to the widefield macro-foci (see Figure 1). The individual 53BP1 super-clusters were organized in sub-clusters of signal tags in the range of about 100 nm matching with the dimensions of 53BP1 SMST nano distribution observed previously in low- and high LET-irradiated cells [29,71]. 

For the MRE11 nuclease, significantly more but smaller SMST super-clusters per nucleus were detected at >19 nano signal tags within a 100 nm search radius. In general, MRE11 clusters were smaller than 53BP1 SMST clusters, which agrees with observations in other human cell lines [72]. On average the minipig nuclei displayed 5.76 (±5.3; P201) and 7.8 (±7.9; P212) MRE11 SMST clusters per nucleus. Control LCL nuclei, on the other hand, presented an average of 9.8 (±6.5) MRE11 SMST super-clusters per nucleus, with the large SD resulting from considerable variation among individual nuclei. The average diameter of the SMST clusters were 0.49 μm (±0.21) and 0.54 (±0.16) for minipig P201 and P212, respectively. In LCL control nuclei the average super-cluster diameter was 0.62 (±0.22) μm, with only the difference between control and minipig 201 being marginally significant (*p* = 0.037). MRE11 signal tag clusters were also observed in undamaged chromatin off the γ-H2AX defined macro-foci, which contributes to higher average cluster values in all samples compared to 53BP1. This effect suggests that not all MRE11 molecules in a nucleus are recruited to the damaged chromatin under the conditions tested here. 

pATM, in contrast to 53BP1 and MRE11, showed very small nanoclusters in the range of 10–60 nm (see below) that resided with the γ-H2AX- or 53BP1-outlined damaged chromatin areas.

### 3.2. Distance Distributions for DDR Factors in Nuclei with Persistent DSB Damage

**Figure 4:**
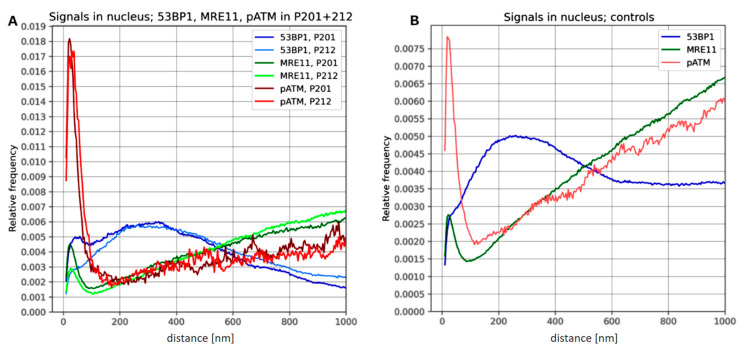
Ripley’s K statistics of 53BP1 (blue), MRE11 (green) and p-ATM (reddish lines) revealing pair-wise nm distance distribution of signal tags in minipig and LCL control nuclei. (**A**) Frequency distribution plots of DDR marker protein single molecule signals in *n* = 25 epidermal keratinocyte nuclei with persistent super-foci from 2 minipigs (P201, P212) 28 days post γ-IR. 53BP1 signal tags show clustering between 200 and 400 nm. In contrast, this frequency drops at larger distances. MRE11 shows a small sharp peak at 10–40 nm with a shallow increase in the keratinocyte nuclei. A similar distribution is seen for pATM with a sharp high peak below 50 nm and a shallow increase at larger distances indicating the absence of pATM molecule signals in the undamaged chromatin of the minipig nuclei. (**B**) LCL control nuclei of (*n* = 25) 30 min post 0.5 Gy X-IR for 53BP1 show a similar clustering like minipig nuclei but constant frequencies at larger distances. MRE11 displays distributions with a steep increase at larger distances, indicating random distribution of MRE11 signal tags throughout the nuclear chromatin. ATM distribution has a peak below 50nm similar to the minipig cells but a steeper increase at larger distances.

Since the three DDR proteins analyzed showed a very different single molecule signal distribution in nuclear chromatin, we investigated the pair-wise distance distributions of the signal tags for the DDR markers at the nanoscale using Ripley’s K-function (Figure 4), which allows the discrimination of defined geometries from random point patterns [29,56].

In epidermal minipig keratinocyte nuclei with persistent DSB damage 53BP1 nano-signal tags showed a small, rather flat distance distribution peak at around 45–50 nm and a 200–400 nm wide super-clustering (Figure 4A), with the latter corresponding to the macro-foci seen in the widefield microscope at mesoscale. A similar, more distinct 53BP1 single molecule nano-distribution was noted in control LCL nuclei 30 min after 0.5 Gy X-irradiation but without a significant distance peak at 35–40 nm (Figure 4B). These data are consistent with a wider 53BP1 distribution as a chromatin factor around DSB regions [28,29,43]. 

MRE11 and pATM, on the other hand, only formed small SMST nanoclusters (<100 nm) with a clear, sharp peak in minipig nuclei (Figure 4A), which was also seen in the control LCL nuclei (Figure 4B). The distribution of pATM single molecule tags in persistent macro-foci formed high sharp peaks, which indicates that the pATM molecules are confined to these structures, suggesting an ongoing DSB damage signaling in the genomic scars of the keratinocytes. 53BP1, on the other hand, showed constant or decreasing values for larger distances, indicating a dearth of 53BP1 molecules outside the persisting macro-foci of minipig cells. At larger distances MRE11 and pATM showed increasing values with a different slope, reflecting their random distribution in undamaged chromatin regions. pATM and MRE11 signal distance curves in LCL control nuclei showed a steep linear increase at larger distances (Figure 4B), indicating the random distribution of these molecules throughout undamaged chromatin of nuclei with freshly induced simple DSBs. The scarcity of pATM SMST signals in undamaged chromatin is reflected by more rugged lines at larger distances (Figure 4). The shallow increases of MRE11 and pATM at greater distances in the nuclei of minipig keratinocytes (Figure 4A), compared to the steep increases in the LCL controls, indicate the predominant localization of the investigated DDR protein nano-tags within persistent foci of minipig keratinocytes.

### 3.3. Persistent Damage Regions Are Enriched for DDR Markers

**Figure 5:**
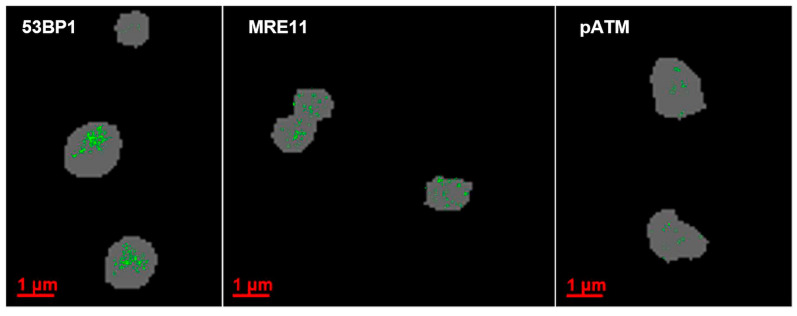
Regions of interest (gray) outlining γ-H2AX macro-foci in nuclear chromatin (black). The green dots within the ROIs display single molecule tags of the indicated markers, with the intensity and size reflecting the frequency of nano signal tags at a particular spot.

To further investigate the local DDR factor organization within persistent DNA damage regions, we used γ-H2AX macro-foci recorded in widefield images under the SMLM microscope and drew ROIs defining these. Regions underlying γ-H2AX ROIs were then subjected to single molecule signal analysis for 53BP1, MRE11, and pATM (Figure 5). It is of note that due to diffraction the γ-H2AX ROIs can be oversized relative to SMLM cluster sizes. 

**Figure 6:**
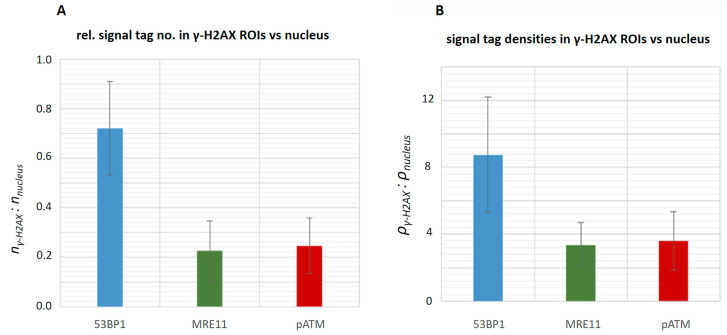
Relative marker distributions in minipig keratinocyte nuclei (**A**) Relative numbers of 53BP1, MRE11 and pATM signal tags within γ-H2AX ROIs vs. all 53BP1, MRE11 and pATM signal tags. (**B**) Densities of 53BP1, MRE11 and pATM signal tags within ROIs of γ-H2AX super-foci relative to that in the surrounding undamaged nuclear chromatin.

Analysis of the Ripley pair-wise distance-frequency curves in the γ-H2AX ROIs and comparison with the corresponding curves for the whole nucleus revealed that the 53BP1 peak is strongly associated with the γ-H2AX ROIs, which is reflected in more than 70% of the 53BP1 signals being located within the γ-H2AX ROIs (Figure 6A). The small 53BP1 clusters represented by the peak at about 50 nm, especially in animal P201 (Figure 4A), could not significantly be associated with the γ-H2AX ROIs. In contrast to that, the MRE11 and pATM peaks (Figure 4) were strongly related to γ-H2AX ROIs, with 23% of MRE11 signal tags and 25% of the pATM signal tags being located inside the γ-H2AX ROIs (Figure 6A).

To further investigate the above results, we analyzed the densities of nano signal tags of the respective DDR proteins within γ-H2AX-positive macro-foci (Table 2) and in the surrounding nuclear chromatin (Figure 6B). 53BP1 signal tags were most abundant in persistent γ-H2AX macro-foci (ROIs) with 8.7-fold (±3) enrichment relative to undamaged keratinocyte chromatin. The MRE11 nuclease was 3.4-fold (±1.3) enriched in persistent macro-foci, as was the active apical kinase pATM (3.6-fold ± 1.8). pATM signal tags were scarce and focally restricted to the γ-H2AX-defined macro-foci, they were rarely seen in undamaged keratinocyte chromatin (Figure 6). 

**Figure 7:**
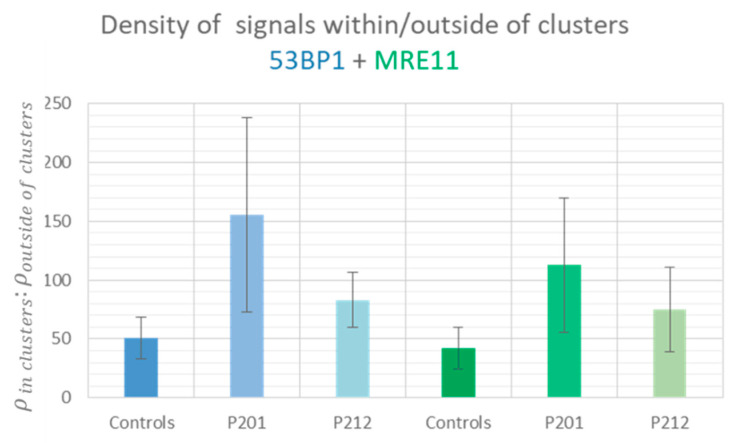
Relative average densities of 53BP1 (blue bars) and MRE11 (green bars) signal tags within γ-H2AX-outlined macro-foci relative to their density in the surrounding undamaged nuclear chromatin in minipig epidermal keratinocytes and irradiated LCL control nuclei.

In contrast to the minipig cells, the macro-foci in LCL controls with 0.5 h young DSBs showed a lower density of DDR markers relative to undamaged chromatin of the same nuclei (Figure 7).

The results described above indicate a preferential accumulation of the analyzed DDR proteins within the γ-H2AX-outlined macro-foci, which is typical for ongoing repair; 53BP1 overlaps the γ-H2AX ROIs, while small clusters of MRE11 and pATM are integrated into the γ-H2AX-tagged chromatin regions. This was also seen in DSB regions of LCL control nuclei 30 min after irradiation and is in agreement with results obtained in other cell types (see, e.g., [29,61,72,73]).

### 3.4. Persistent Homologies among Nano-Organization Patterns of Damage Foci Reveal Radiation Quality

To test for topological similarities or differences in the spatial organization of the persistent DNA damage macro-foci and to further compare them to high LET-induced damage, we next analyzed persistent homologies concerning 53BP1 and MRE11 nano distribution in irradiated minipig keratinocytes and freshly formed macro-foci of LCL nuclei, and compared these with data on the nano distribution of DDR markers in high LET alpha particle-induced damage foci tracks in human lymphocytes [29]. 

**Figure 8:**
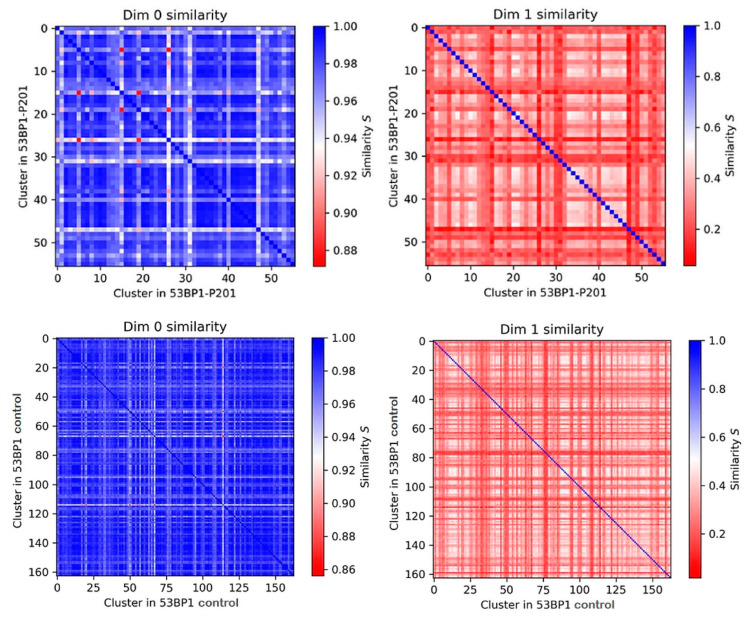

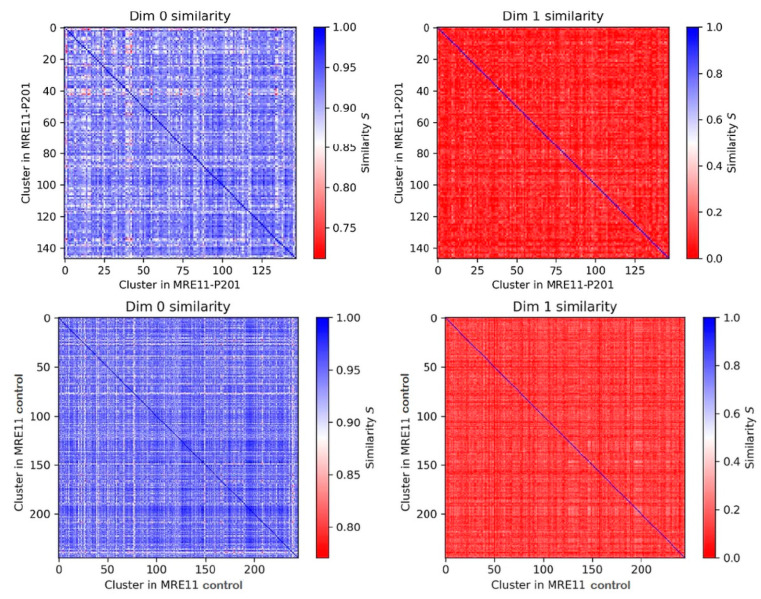
Examples of similarity heatmaps for topological features of 53BP1 and MRE11 signal tag nanoclusters in γ-H2AX damage areas in minipig (P201) keratinocyte nuclei and LCL control cell nuclei. Note, that in general, the similarity of the components is overestimated (dim 0), while the similarity for the holes (dim 1) is much lower.

Persistent homology analysis [62] was performed for dimension 0 (components; dimensions of the distance patterns of nodes defined by the single-molecule signals and their nanoclusters) and dimension 1 (holes; area dimensions between single molecule signals and their clusters) for 53BP1 and MRE11. For comparison of topological similarity among the nanocluster distribution in the super-foci of the different animals and cells independently of the cell nucleus, we calculated the Jaccard indices [70] and visualized the comparisons in appropriate heatmaps [56,62,68]. The results revealed a high topological similarity among the nanocluster distribution in the super-foci of photon-irradiated cells, i.e., minipig keratinocytes and LCL cells. Figure 8 displays representative heatmaps for animal P201 and the LCL control. It should be noted that the similarity of the components is generally overestimated, while the similarity of the holes is much lower (see for comparison [62,63]). In that way, the heatmaps for dimensions 0 and 1 (Figure 8) can be assumed as the upper and lower limit of similarities, respectively.

**Figure 9:**
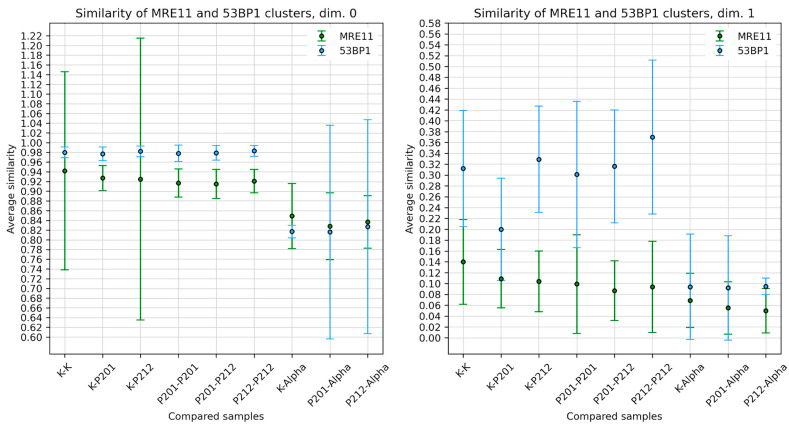
Average similarities for dimensions 0 and 1 between the nanocluster topologies of 53BP1 (blue lines) and MRE11 (green lines) among the different probes and between themselves (K, clusters in control LCL nuclei; P, in minipig nuclei). While the topological similarities of the nanocluster distribution of MRE11 and 53BP1 are similar in the damaged areas of low LET irradiated minipig keratinocyte and LCL nuclei, the former and alpha-particle damage tracks (data taken from [29]) clearly show different topological features in the damaged chromatin for dim.0. For dimension 1 this only the case for 53BP1.

Figure 9 compares the average similarity values (Jaccard indices) among the different cells and animals investigated. These features were compared to the nano-organization of DSB damage cluster tracks of alpha-irradiated lymphocytes (taken from [29]). For the components (dimension 0), a very high similarity was observed for 53BP1 and MRE11 signal tag nanocluster distributions among the LCL control nuclei and keratinocyte nuclei of both minipig samples. However, the similarity values were reduced when comparing the former with the nano-organization of DDR proteins in high LET α-particle induced DSB damage tracks (Figure 9). For the holes (dimension 1), this effect was only observed for 53BP1, while for MRE11 the similarity values did not change considerably. The similarity values of the hole dimensions (dim 1) for 53BP1 were relatively equal for the LCL control and among the animals P201 and P212, suggesting a similar chromatin organization among the low LET-induced damage regions. 

## 4. Discussion

Acute 50 Gy γ-irradiation of Göttingen minipig pig skin rapidly induced massive DNA damage in exposed keratinocyte nuclei, leading to widespread histone H2AX phosphorylation (pan-γ-H2AX) and high numbers of radiation-induced 53PB1 foci [13], as approx. 2000 DSBs will be induced in each nucleus by this acute dose [74]. The subsequent temporal progression of DNA repair led to the loss of the pan-γ-H2AX pattern in favor of numerous γ-H2AX foci in exposed nuclei, which eventually disappeared except for a few persisting focal damage areas per keratinocyte nucleus, with the latter still being present several weeks after the exposure. These persistent foci still contained γ-H2AX, 53BP1 and active pATM DDR proteins 28 days post IR or even later, with the average radiation-induced DDR foci numbers still being more than two-fold increased above control values [13]. It has been suggested that large persistent radiation-induced foci likely reflect the clustering of chromatin regions harboring multiple damage types and unrepaired DSBs [26,35,75,76,77,78]. Persistent DNA damage-related foci have also been observed to accumulate as a consequence of cellular aging [79] and after exposure to high LET radiation [80], and are, furthermore, considered markers of lethal DNA damage (see [36]) or of induced genome instability [28,44]. 

To better understand the chromatin characteristics and the relation to DNA repair processes within such persistent foci, we analyzed the nano-distribution and topological properties of single molecule signals of DDR markers in persistent macro-foci of keratinocyte nuclei of two minipigs 28 days after 50 Gy γ-irradiation. Single-molecule localization microscopy of immunostained paraffin tissue sections showed that the persistent DNA lesions in minipig keratinocytes 28d after irradiation display a non-random organization of the DDR factors. The DNA damage sites contained a high density of 53BP1 nano-signal tags colocalizing within γ-H2AX-tagged widefield macro-foci, while the undamaged nuclear chromatin showed a dearth of 53BP1 single molecule signals.

MRE11 nuclease single molecule tags were enriched and clustered in the persistent macro foci to which activated pATM single molecule signals were confined. This distribution of pATM, the apical kinase in the IR-induced DNA damage response cascades [81], suggests that the DDR in persistent damage foci is constantly driving DSB damage signaling and this even weeks after high dose γ irradiation of the minipig skin. In contrast, freshly induced DSB regions of X-irradiated LCL control cells displayed a similar nano organization of DDR molecules in their focal DSB regions but more DDR molecules in the undamaged chromatin. The nano tag distribution features of the DDR markers were similar in keratinocyte and LCL cell damage foci, suggesting that the two different mammalian cell types respond similarly to low LET irradiation at the structural level. 

High LET particle irradiation, on the other hand, has been observed to cause long-lasting rearrangements in chromatin architecture along the particle trajectories, which appear as nuclear chromatin scars with DNA damage signaling [82,83]. To see whether there are similarities between DSB damage marker distribution among the persistent foci of γ-irradiated minipig skin and α-particle-induced complex DNA damage, we performed persistent homology comparisons [62] between the single molecule nano-distribution patterns in persistent foci and the previously observed in α-particle-induced DNA damage tracks in human mononuclear blood cells [29]. These comparisons clearly separated the DDR the marker nano-distribution in high LET alpha-particle damaged chromatin from the nano-distribution patterns in the low LET-induced persistent macro-foci of the minipig cells and the freshly formed DSB damage foci of human LCL control cells. This suggests that low LET irradiation induces structural similarities in DDR marker nano-distribution among freshly formed and persistent DSB damage chromatin regions in the different mammalian cell types studied. On the other hand, these chromatin arrangements are distinct from the more severely damaged and rearranged chromatin induced by high LET irradiation [29,82]. Still, 30% of the DSBs induced by low LET are considered to be complex [28], so it may be that such complex DNA damage accumulates over time in the persistent foci, but the overall damage distribution within persistent foci seems to be less dense and distinct from the more concentrated damage after high LET alpha irradiation. It will thus be of interest to learn more about the chromatin nano-organization in in vivo irradiated tissues weeks after the exposure and to study the organization of other DNA damage types and the molecular responses and physiological alterations in cells with persistent foci. 

While it is clear that there are cell type-specific responses to irradiation at the 3D genome level [84,85], our observations suggest that the persistent genomic lesions in irradiated skin continuously recruit DDR proteins, possibly in an attempt to instigate repair at the focal chromatin regions containing non-repairable complex DNA damage and/or containing altered chromatin structures [28,86]. Eventually, cells may even extrude the damaged DNA regions from the nucleus, e.g., as micronuclei [87], to consign them to degradation. It is clear that the latter will be a source of genome mutation that may lead to genomic instability, senescence, cancer, or cell death [88,89]. Furthermore, it seems possible that continuous DNA damage signaling in response to irreparable DNA damage in persistent foci may trigger the senescence-associated secretory phenotype (SASP) [52,53] that may negatively influence tissue repair of irradiated skin [11,90], and thereby contribute to the known radiation-induced skin injury that is associated with impaired healing and chronic wound recurrence [11,91].

## Figures and Tables

**Table 1:** Antibodies, dilutions and sources.

Antibodies	Dilution Used	Source
Primary antibodies		
- mouse anti-γ-H2AX mab (JB301)	1:500	Sigma-Aldrich, Taufkirchen, Germany
- rabbit anti-53BP1	1:500	Abcam, Cambridge UK
- rab anti pATM pS1981 mab (EP1890Y)	1:400	Abcam, Cambridge UK
Secondary antibodies		
- Goat anti-mouse Alexa 488	1:600	Dianova, Hamburg, Germany
- Donkey anti-rabbit-Cy3	1:800	Dianova, Hamburg, Germany

Single Molecule Localization Microscopy (SMLM). SMLM was performed on an in-house-made instrument as described earlier [29,42] and nuclei of 30 to 60 field-of-view regions per preparation were recorded with a 100x/NA 1.46 oil plan apochromatic objective lens (Carl Zeiss Microscopy, Göttingen, Germany) on tissue sections immunostained for the proteins 53BP1, γ-H2AX, MRE11 and pATM. Fluorophore counts and image quality (with corresponding widefield images) were considered as criteria for sorting and removal of artifacts and outliers, rendering 25 nuclei per experiment, preparation and tissue type. Pre-tests showed that 90% of the laser power of the 561 nm laser, corresponding to 198 mW, produced the most distinct signals discriminating the DSB foci from the background noise.

**Table 2:** Density and number of signal tags within γ-H2AX super-foci.

Tissue (DDR Protein)	Average Number of Signals ± SD in 1/µm^2^
LCL-control (53BP1)	709 ± 99
P201 (53BP1)	708 ± 188
P212 (53BP1)	689 ± 126
LCL-control (MRE11)	1941 ± 440
P201 (MRE11)	2156 ± 700
P212 (MRE11)	1852 ± 493

## Data Availability

Data are available on request from the authors.
